# Bronchial cast hiding a lung cancer

**DOI:** 10.1186/2049-6958-7-43

**Published:** 2012-11-13

**Authors:** Marcello Migliore, Nicola Ciancio, Riccardo Giuliano, Giuseppe Di Maria

**Affiliations:** 1 Thoracic Surgery, University of Catania, Catania, Italy; 2 Respiratory Medicine, University of Catania, Catania, Italy; 3 Department of Surgery, Thoracic Surgery, Policlinico Hospital, Via S. Sofia 78, Catania, Italy

**Keywords:** Airway obstruction, Bronchial cast, Broncoscopy, Haemoptysis, Lung cancer, NSCLC

## Abstract

A 70-year-old man was admitted for severe hypoxia, haemoptysis and cough. Chest-X-ray and CT-scan indicated a right-lower-lobe collapse. Bronchoscopy showed its occlusion by whitish dense mucus. Aspiration revealed a Bronchial Cast (BC) and a stenotic and inflamed orifice of the right-lower-lobe-bronchus which was biopsied.

Histopathologic examination of BC showed fibrin with lymphocytes and neutrophils, and, surprisingly, also the presence of lung cancer. Although the association between BC and benign, myxoid-soft-tissue, tracheobronchial tumors has been described, the association with lung cancer has not previously been reported, and it remains unclear whether it is causal or casual.

## Background

Bronchial Cast (BC) is a complication of uncertain pathogenesis and is often associated with diseases involving mucus hypersecretion. The organization of the mucus into the shape of the tracheobronchial tree, also known as plastic bronchitis, occasionally leads to acute respiratory failure.

BC formation is not very rare, but the aetiology is rarely evident despite a long list of differential diagnoses [1].

The association of BC with a hidden lung cancer has not previously been described.

## Case presentation

**Figure 1:**
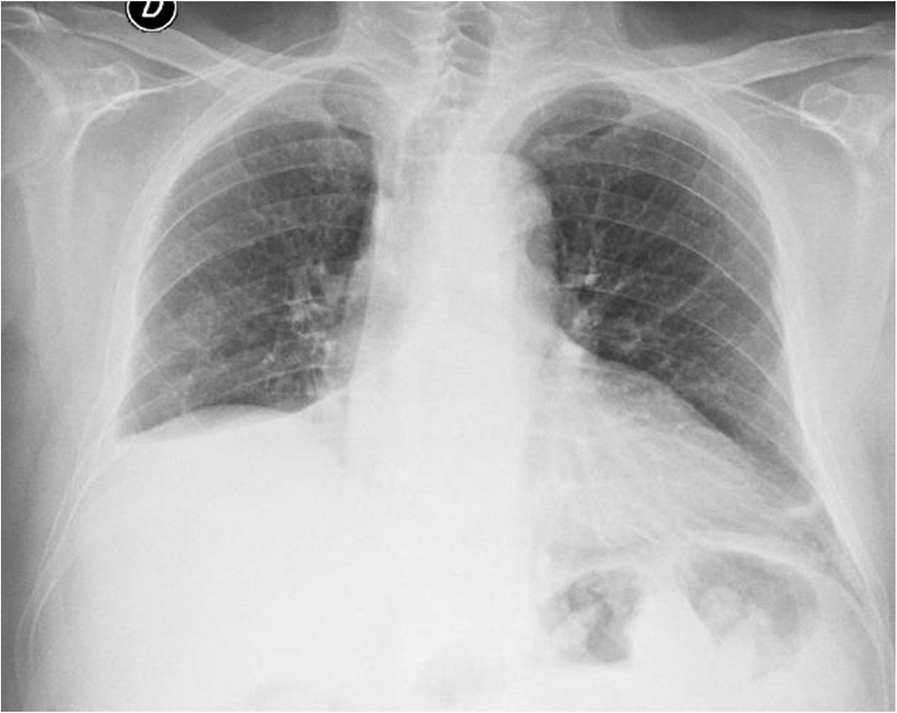
Frontal chest radiograph showing right lower lobe collapse.

A 70-year-old man with 2-month history of chest trauma presented with dyspnea, hemoptysis, and productive dense cough. He was an ex smoker (30 cigarettes/day). The patient had suffered from recurrent chest infections in the last two months. He was admitted because of desaturation with hypoxia, and arterial blood gas analysis evidenced: pH 7.48, pCO_2_ 39 mmHg, pO_2_ 49 mmHg and SpO_2_ 87%. Chest X-ray showed an opacity in the right-lung base (Figure 1). The CT scan confirmed the presence of the right lower lobe collapse. Sputum examination identified *Citrobacterfreundii* and *Staphylococcus aureus.* Symptoms did not improve after 6 l/min oxygen and intravenous antibiotic therapy.

**Figure 2:**
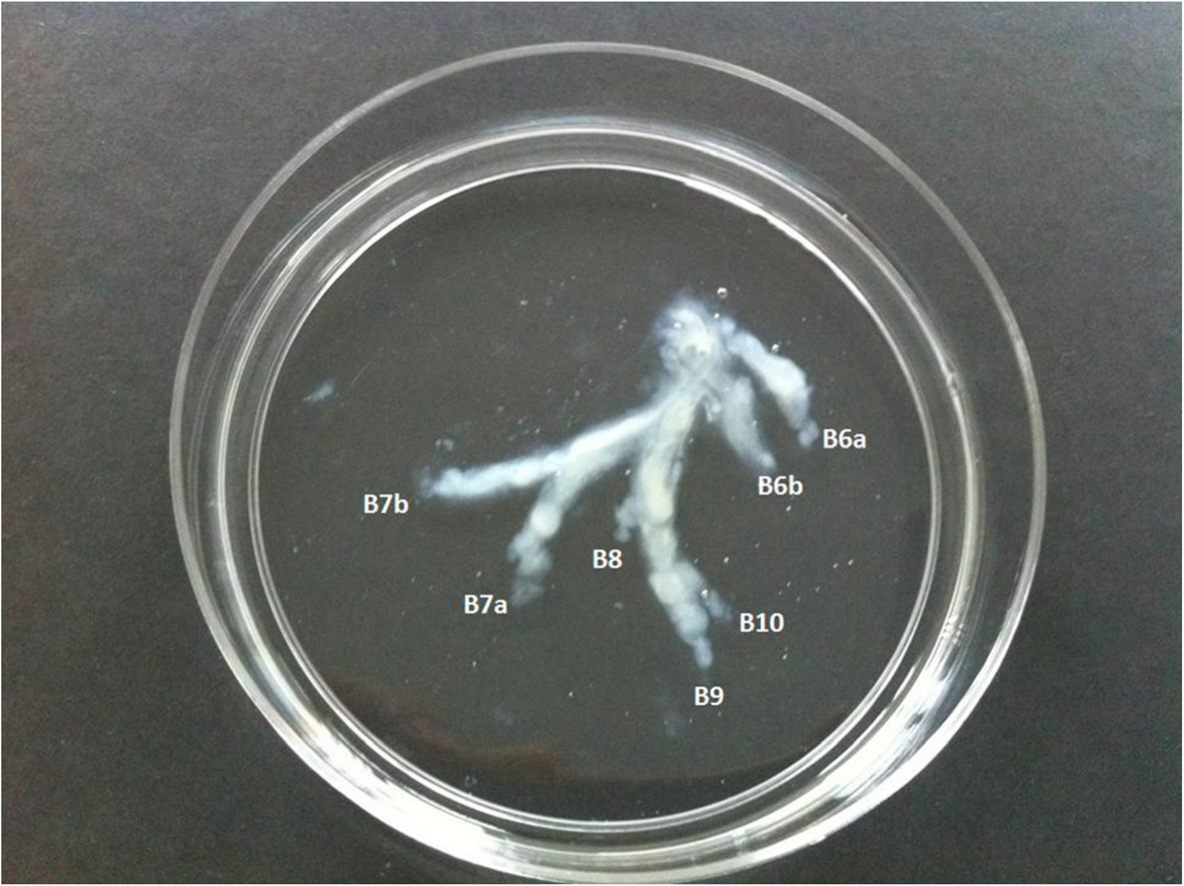
**Macroscopic whitish, rubbery bronchial cast of the RLL floating in normal saline.** B6a: apical medial, B6b: apical superior, B7a: paracardiac lateral, B7b: paracardiac medial, B8: anterior basilar, B9: lateral basilar, B10: posterior basilar.

Bronchoscopy was subsequently performed to evaluate possible alterations of bronchial airways. The orifice of the right lower lobe was occluded by a white phlegm with dense consistency. It was too dense to be aspirated through the bronchoscopic channel and therefore it was removed stuck on the distal end of the bronchoscope. When it was immersed in normal saline the “phlegm” floated in the solution, and a BC appeared (Figure 2).

The orifice of the apical segment of the lower lobe was iperemic, edematous and stenotic, and biopsies wereperformed. Surprisingly, although the BC showed findings of eosinophilia and neutrophilic infiltration, the bronchial biopsy showed the presence of non small cell lung cancer.

Gross examination of the BC showed a whitish, rubbery cast in the shape of the bronchial tree (Figure 1). Histopathologic examination of the BC revealed mucus and fibrin with enmeshed red blood cells, lymphocytes, few neutrophils, and foamy macrophages. Results of smears and cultures for fungi and mycobacteria were negative.

## Discussion

Casts may shape at any point in the tracheobronchial tree, with remarkable examples of casts bearing perfectly defined bronchial branches. Bronchial casts are classified as type I (cellular) or type II (acellular) [2]. Type I casts occur in the lung inflammatory framework and consist mainly of fibrin and inflammatory cells. Type II casts are seen more commonly in patients with a history of palliative surgery for congenital cyanotic heart disease, and consist of mucin without inflammatory infiltrate. In our patient the occurrence of mucus and fibrin with lymphocytes, few neutrophils, and foamy macrophages clearly suggests that it was a type I BC. It was surprising to find an underlying lung cancer, really discovered by chance.

Bronchial casts may have different origin and in our case it may be considered as a type of plastic bronchitis given that it had moulded to the figure of the right lower lobe.

Bronchial cast formation is not very rare, but the aetiology is rarely evident despite the long list of differential diagnoses. The organization of the mucus into the shape of the tracheobronchial tree, also known as plastic bronchitis, occasionally leads to acute respiratory failure. To the best of our knowledge the association of BC with a hidden lung cancer has not previously been described.

Plastic bronchitis is usually associated with diseases that involve bronchial hypersecretion, like pneumonia, diphtheria, tuberculosis, asthma, inhalation of foreign bodies, allergic bronchopulmonaryaspergillosis, pulmonary hemorrhage, bronchiectasis, cystic fibrosis, chronic bronchitis, cardiac valvular abnormalities, and amyloidosis [1]. When it cannot be related to any of these entities, it is considered idiopathic. It is therefore clear that without the bronchial biopsy, the cast removed by us would have been considered idiophatic.

There are no standardized treatments for BC, likely due to the small number of cases, therefore the evidence remains anecdotal and not irrefutable [3]. The reported treatments include bronchodilators, antibiotics for bacterial infection, hydration, chest physiotherapy, postural drainage, and bronchoscopic removal of casts. Kruger et al. point out that acute respiratory failure, presenting with wheezing and thoracic air leakage refractory to standard asthma therapy, should raise the suspicion of cast bronchitis, and suggest that urgent bronchoscopy or VATS be performed [3-6].

## Conclusions

Although the association between BC and a benign myxoid soft-tissue tracheobronchial tumor has been described [6], the association with a lung cancer has not previously been reported, and it remains unexplained whether this association is causal or casual.

Our finding demonstrates that the presence of a bronchial cast imposes the necessity to perform a target biopsy to exclude the potential concomitance of a hidden lung cancer.

## Consent

Written informed consent was obtained from the patient for publication of this Case report and any accompanying images. A copy of the written consent is available for review by the Editor-in-Chief of this journal.

## Competing interests

The authors declare that they have no competing interests.

## Authors’ contributions

MM carried out the videobroncoscopy with biopsy and the final operation, conceived and drafted the manuscript. NC and RG participated in the design of the study. GD participated in the design and coordination of the study and helped to draft the manuscript. All authors read and approved the final manuscript.
